# High prevalence of Duck Hepatitis B virus-associated coinfection in Southwest China

**DOI:** 10.1371/journal.pone.0324682

**Published:** 2025-06-16

**Authors:** Xiaoming Lin, Lizhen Gong, Yajia Gou, Yi Liu, Sai Mao, Shun Chen, Mafeng Liu, Dekang Zhu, Mingshu Wang, Renyong Jia, Shaqiu Zhang, Ying Wu, Juan Huang, Bin Tian, Qiao Yang, Xinxin Zhao, Anchun Cheng, Xumin Ou

**Affiliations:** 1 Institute of Veterinary Medicine and Immunology, Sichuan Agricultural University, Wenjiang, Chengdu, Sichuan, China; 2 Engineering Research Center of Southwest Animal Disease Prevention and Control Technology, Ministry of Education of the People’s Republic of China, Sichuan Agricultural University, Chengdu, Sichuan, China; 3 Key Laboratory of Animal Disease and Human Health of Sichuan Province, Sichuan Agricultural University, Chengdu, Sichuan, China; 4 Key Laboratory of Agricultural Bioinformatics, Ministry of Education, Chengdu, Sichuan, China; Universidad Cooperativa de Colombia, COLOMBIA

## Abstract

Currently, five types of duck hepatitis viruses have been documented, and they are all associated with liver disorders. However, the prevalence of their coinfections involving these viruses remains largely uncertain. Herein, we screened the prevalence of the five types of hepatitis viruses from A to E in 143 samples of diseased duck livers during 2019–2021 in Southwest China. We found the highest infection ratio (86.01%, 123/143) of duck hepatitis B virus (DHBV) among all five types of hepatitis viruses. Importantly, a large portion of DHBV-associated coinfections were identified, with 52.85% (65/123) co-infected with Duck Hepatitis A virus (DHAV), 39.84% (49/123) with tentative Duck Hepatitis D virus (DHDV), and 34.96% (43/123) with Duck Hepatitis E virus (DHEV), respectively. Interestingly, a positive correlation between the DHBV-positive rate and the infection rates of the other co-infected hepatitis viruses was revealed, suggesting the importance of DHBV in duck hepatitis virus co-infection events. To understand the situation of bacterial secondary infection, the prevalence of bacterial infection was simultaneously screened using standard 16S rRNA PCR, and hepatitis virus-associated bacterial infections were observed. Collectively, these findings revealed a high prevalence of DHBV-related coinfections and its association with the coinfection of the other duck hepatitis viruses and bacteria. In the future, it is important to study the impact of DHBV co-infection events on disease severity, thereby evaluating the necessity of vaccine development for DHBV.

## Introduction

Currently, all five types of duck hepatitis viruses have been separately documented in diseased duck livers. These include duck hepatitis A virus (DHAV), duck hepatitis B virus (DHBV), duck hepatitis C virus (DHCV), duck hepatitis D virus (DHDV), and duck hepatitis E virus (DHEV). The infections of DHAV and DHBV are substantiated. However, the other three hepatitis viruses were only verified by viral sequences and were thus tentatively named in a nomenclature system we recently proposed [1,2]. The disease burden caused by DHAV is high; it is one of the five most fatal agents in ducklings [3,4]. The ducklings infected with DHAV are highly fatal and cause ~90% of mobility and mortality, whereas the mature ducks infected by the virus usually remain asymptomatic. The infected ducklings are characterized by a severe form of acute viral hepatitis with typically clinical signs such as opisthotonos, hemorrhages, and liver necrosis [5]. The DHAV has now been classified into genotypes 1–3. Genotype 1 of DHAV is distributed globally, while the other two genotypes, especially genotype 3, are mainly reported in Asia [6]. A meta-analysis of 689,549 samples from 14 provinces of China reported that the positive rates for DHAV-1 and DHAV-3 were 38% and 49%, respectively [7].

Unlike the DHAV, DHBV infection typically causes no obvious liver damage, and the infected ducks generally remain healthy. Thus, there is little pathogenic information related to DHBV infection, and generally being neglected in the duck industry or veterinary studies [2,8]. However, the positive rates of DHBV infections are much higher than we expected. For example, a recent DHBV epidemic survey across 54 waterfowl farms revealed an overall positive rate of 52.2% in duck populations [9]. Similar to this survey, another independent investigation reported a similar result, with a 58.6% DHBV-positive rate among 29 duck farms [10].

For the other three types of hepatitis viruses (from DHCV to DHEV), little is known about their virological and pathogenic characteristics. DHCV infection was first reported in 2019, and some primary investigations showed the presence of DHCV RNA in poultry livers from meat markets [11]. Recent epidemiological observations indicate that 69.7% of ducks were tested positive for DHCV infections [12]. For potential DHDV, only HDV-like sequences have been found so far, with little known about its pathogenicity, clinical manifestations, or epidemiology [13,14]. Avian HEV, a member of Orthohepevirus B, was originally isolated from chickens suffering from “big liver and spleen disease” (also known as hepatitis-splenomegaly syndrome) [15]. Key symptoms include pale or white combs with red edges, red ascites in the abdomen, poor physical condition, degeneration of the ovaries and oviducts, and liver and spleen enlargement [16]. The prevalence of avian HEV is not limited to chicken but fairly in ducks; however, one survey found that 9 out of 30 ducks were positively infected with the virus [15]. Of note is that the infection of these three types of hepatitis viruses in ducks was pinpointed by genetic evidence and viral RNA identity. These findings, however, suggest the presence of these three viruses in the duck populations.

The above-separated observations fully indicated a possibility of circulating all these five types of hepatitis viruses in duck populations [2]. However, coinfections of these viruses have been largely neglected, and no systematic investigation on their coinfection patterns has been conducted so far. To this end, we systematically collected liver samples from diseased ducks from Southwest China. In the present study, we found that DHBV infection was highly prevalent, and its related coinfections were commonly observed. This finding discovered an emerging problem of DHBV-related co-infections and thus requires more investigations to evaluate the functional impact of DHBV infection on the co-infected hepatitis virus, as well as on disease severity.

## Materials and methods

### Ethics statement

This study was conducted in strict adherence to the ARRIVE (Animal Research: Reporting of In Vivo Experiments) guidelines. The experimental protocols involving animals were reviewed and approved by the committee on operational experiment guidelines and animal welfare of Sichuan Agriculture University, China.

### Samples

Liver samples were collected by the Research Center of Avian Disease, Sichuan Agricultural University, from 2019 to 2022. Most of the samples were collected from farms surrounding Chengdu, Sichuan, China, where farmers sent the diseased or dead ducks to our lab for pathogen diagnosis. The ducks sent to us were sampled by researchers following strict aseptic procedures. All liver samples were immediately sampled from the deceased ducks. In total, we collected 143 liver samples, summarizing the clinical data in Table 1.

**Table 1:** Clinical characteristics of liver samples.

Characteristics	Numbers (n = 143)	Percentage
**Geographical distribution**		
Sichuan province	103	72.00%
Shanxi province	2	1.40%
Hubei province	9	6.30%
Jiangxi province	7	4.90%
Unknown	22	15.40%
**Age**		
Yong(<=28days)	70	49.00%
Adult (>28 days)	43	30.10%
Unknown	30	21.00%
**Host species**		
duck	135	94.40%
goose	8	5.60%
**Death or alive**		
Dead	110	76.90%
Dying	9	6.30%
Live	24	16.80%
**Manifestation of liver**		
Hyperemia	39	27.30%
Hepatic fibrosis	27	18.90%
Brittle	21	14.70%
Yellow liver	15	10.50%
Other	12	8.40%
Normal	3	2.10%
Unknown	40	28.00%

### DNA/RNA isolation and Reverse transcription.

DNA and RNA from liver tissues were extracted using the Takara Mini BEST Viral RNA/DNA Extraction Kit Ver.5.0 (Cat#, 9766), following the manufacturer’s instructions. The RNA concentration of the samples was quantified to 400 ng for reverse transcription using the Nanodrop One instrument. The Takara Prescript™ RT reagent Kit (Cat#, RR036) was utilized for cDNA synthesis. The RT reaction mixture was incubated at 37°C for 15 minutes, followed by inactivation at 85°C for 5 seconds.

### Primer design

Genomic sequences of all five duck hepatitis viruses were downloaded from the NCBI Virus database (https://www.ncbi.nlm.nih.gov/labs/virus/vssi/#/). The NCBI Primer–BLAST tool (https://www.ncbi.nlm.nih.gov/tools/primer-blast/) was exploited to design specific primer pairs while keeping all the primer pairs having the same Tm value (60 ± 2°C) and similar PCR product length (~250 bp). The selected primers were further validated by another round of BLAST that excluded any unspecific amplification of the duck host genes. Before being used in clinically collected samples, the final primer pairs were analyzed by melt curves (a method to check if a single PCR product was amplified) when a compatible qPCR procedure was finished (see below detailed method). This bioinformatic analysis and experimental validation guarantee the specificity and compatibility of qPCR quantification, which was also confirmed by sequencing of these qPCR amplicons.

### qPCR

qPCR was performed on a CFX96 Real-Time PCR Detection System (Bio-Rad Laboratories, Hercules, CA, USA) using Taq Pro Universal SYBR qPCR Master Mix (Cat#, Q712). According to the qPCR manual, the reaction consisted of a total volume of 10 μl, including 5 μl of the Master mix, 2.6 μl of ddH_2_O, and 0.2 μl of each upstream and downstream primer (10 pmol/μl). The PCR cycling conditions were as follows: a denaturation step at 95°C for 30 seconds, followed by 45 cycles of 95°C for 10 seconds, 60°C for 10 seconds, 72°C for 30 seconds, with a final cycle at 95°C for 15 seconds and 60°C for 5 seconds. To make the qPCR reaction compatible with all hepatitis viruses, the length of the PCR product was designed within 200–300 bp with melting temperatures around 60 ± 1°C both upstream and downstream primers. The primer sequences are listed in the S1 Table.

### Bacterial screening

A pair of 16s RNA targeting primers was used for bacterial screening. The forward primer sequence (27 F) is 5’TTTAAGAGTTTGATCCTGGCTCAG3’; the reverse primer sequence (1492R) is 5’GGTTACCTTGTTACGACTT3’ [13]. PCR amplification was carried out using the Takara Premix Taq™ (Takara Taq™ Version 2.0 plus dye) reagent, with the following amplification conditions: step 1, denaturation, 30 seconds at 94°C; step 2, annealing, 30 seconds at 55°C; step 3, extension, 90 seconds at 72°C; After 30 cycles, the PCR amplicons were run by electrophoresis. The electrophoresis results are in S1 Fig. The raw gel electrophoresis images related to S1 Fig are provided in the Supporting Information (S1 raw images.pdf).

### Data processing

Positive hepatotropic virus samples were identified based on the Ct values and were summarized in the S2 Table. According to qPCR methodology and practices, a CT value greater than 35 is generally considered a negative cut-off value, which was also the standard of the COVID-19 test [17]. Since our lab successfully isolated and cultured the DHAV-3 virus from clinical samples [18], we determined the standard curve following a previously established protocol (S2 Fig). Based on the standard amplification curve and variation of Ct values for DHAV-3, a negative cut-off Ct value was set as 37. Bacterial positive samples were determined by positive bands in gel electrophoresis.

### Coinfection and association analysis

The coinfection status is determined by the simultaneous presence of two or more pathogens in the same samples. For example, the DHAV/DHBV coinfection ratio is calculated by dividing the number of positive samples with both DHAV and DHBV by the total number of samples. To understand how having one type of hepatitis virus might affect getting other types of viruses, we compared the numbers of samples co-infected by this virus and non-coinfected samples without this virus. This association analysis allows us to understand the impact of the co-infected viruses.

### Statistical analysis.

All figures were plotted with GraphPad. The students’ T-test was used for statistical analysis. A P-value less than 0.05 was considered significant.

## Results

### The infection rate of five types of hepatitis viruses and bacteria in duck livers

**Fig 1:**
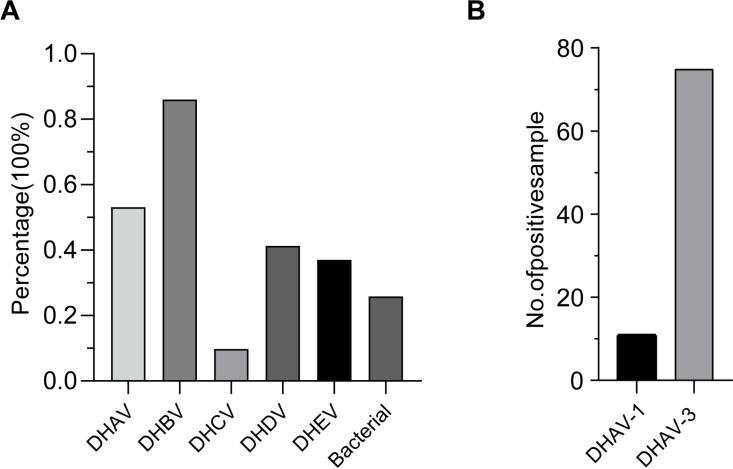
Infection rates of five types of duck hepatitis viruses and bacteria in liver samples. (A) Positive infection rates of five types of hepatitis viruses (from A to E) and bacteria (n = 143). (B) The positive sample numbers of DHAV-1 and DHAV-3.

Before knowing coinfection statuses among different duck hepatitis viruses, it is better to understand their individual infection rates. As screened, we found that the DHAV infection rate is 54.55% (78/143) among multiple farms in the southwest region of China (Fig 1A), with 11 DHAV-1 and 75 DHAV-3 positive liver samples (Fig 1B). Notably, there are 8 liver samples co-infected with both DHAV-1 and DHAV-3. Surprisingly, the infection rate of DHBV is remarkably high at 86.01% (123/143), as demonstrated (Fig 1A). For the others, the DHCV, DHDV, and DHEV infection rates are 9.79% (14/143), 41.26% (59/143), and 37.06% (53/143), respectively (Fig 1A). To understand the situation of bacterial secondary infection, the infection status of bacteria from the same batch of liver samples was simultaneously screened. Based on our data, 25.87% (37/143) of bacterial-positive samples were found (Fig 1A and S1 Fig).

### Identification of high DHBV-associated coinfections in diseased duck livers

**Fig 2:**
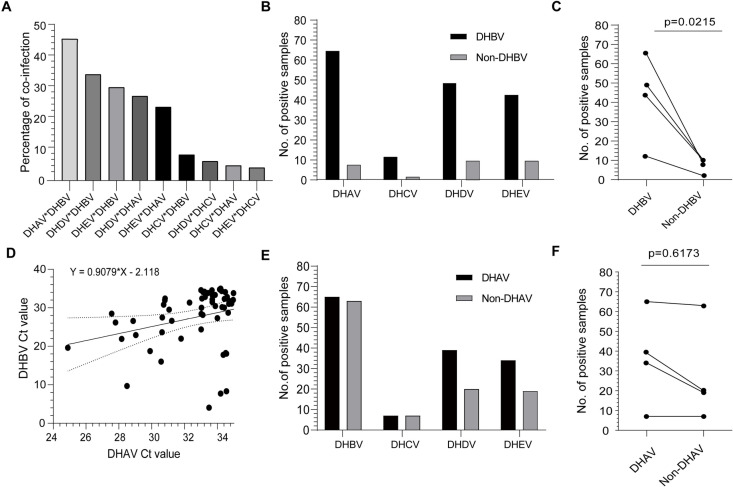
Coinfection analysis between different duck hepatitis viruses. **(A)** Coinfection rates between two out of the five types of hepatitis viruses. **(B)** The positive sample numbers of co-infected hepatitis viruses with or without DHBV. **(C)** The impact of DHBV infection on their coinfection with other hepatitis viruses. The Students’ T-test was used for statistical analysis. **(D)** Correlation analysis between DHBV and DHAV coinfection. Ct values were used, and the Student’s T-test was used for statistical analysis. **(E)** The positive sample numbers of co-infected hepatitis viruses with or without DHAV. **(F)** The impact of DHAV infection on their coinfection with other hepatitis viruses.

Next, we conducted a dual coinfection analysis and successfully identified nine distinct co-infection patterns among the five hepatitis viruses (Fig 2A). Interestingly, we unexpectedly found that DHBV-related coinfections were much more common, for example, reaching 45.45% between DHAV and DHBV infection (Fig 2A). For the other hepatitis viruses coinfected with DHBV, the coinfection rates between DHBV and DHDV were 34.27% (49/143), and 30.07% (43/143) for DHBV and DHEV coinfection (Fig 2A).

Of note, all liver samples were collected from diseased ducks. Thus, the coinfection rates among these duck hepatitis viruses are possibly much higher than those in general duck populations. Despite this, matching each virus to liver samples in our dataset enables us to study how different hepatitis viruses co-infect ducks. Although other coinfection patterns exist, the coinfections between DHBV and the others are much higher than those of the other coinfection pairs (Fig 2A). This prompted us to investigate if DHBV infection is associated with the disease of other hepatotropic viruses (Fig 2B). As tested, DHBV infection significantly increased the infection possibility with the other four duck hepatotropic viruses (p = 0.0215) (Fig 2C). Due to the high coinfection rate of DHBV and DHAV, a correlation analysis between their CT values was conducted (Fig 2D). A clear linear association between DHAV and DHBV infection was also observed as tested.

Additionally, we examined the association between DHAV infection and the other four duck hepatitis viruses (Fig 2E). While there is a low tendency for high coinfection between DHAV and other hepatitis viruses, it did not reach statistical significance (p = 0.6173) (Fig 2F). We also performed a similar association analysis on DHDV or DHEV-related coinfections. However, a significant correlation between DHDV (p = 0.7986) infection rates and other hepatitis viruses was not observed, and neither was the case for DHEV (p = 0.4731) (S3 Fig). A similar analysis was not performed because there were few DHCV-positive samples.

### Bacterial secondary infection is likely associated with the infections of duck hepatitis viruses.

**Fig 3:**
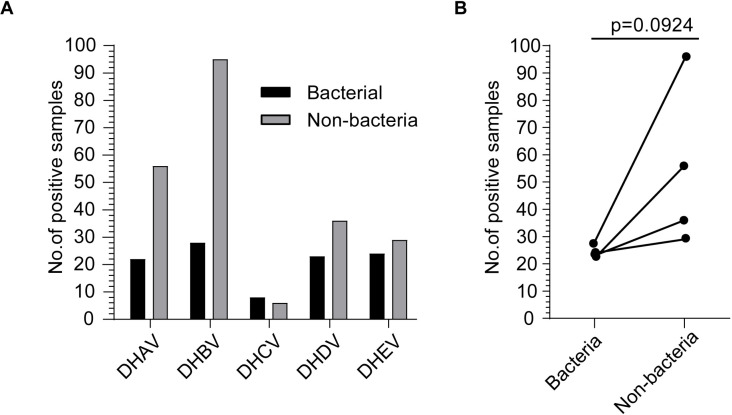
The association between bacterial infection and hepatitis virus infection. **(A)** The positive sample numbers of five types of hepatitis viruses with or without bacterial infection. **(B)** The impact of bacterial infection on their coinfection with five types of hepatitis viruses. The Student’s T-test was used for statistical analysis. DHCV was excluded due to the small number of positive samples.

Because bacterial secondary infection is a major consequence of viral infection, we indeed observed that many samples were infected with not only duck hepatitis viruses but also bacteria (Fig 3A). Specifically, all five types of hepatitis viruses can coexist with bacterial infection in some liver samples. Next, we compared the infection numbers of five hepatitis viruses with or without bacterial infection in all liver samples. Interestingly, our analysis showed that the bacterial infection seems to be a negative regulator for the coinfected hepatitis viruses, although not reaching a statistical significance (p = 0.0924) (Fig 3B).

## Discussion

DHBV not only has a high infection rate in China but also shows a 47% prevalence in farms in other countries, such as South Africa [9,10,19]. In present study, we discovered an extremely high magnitude of DHBV-positive infection (Fig 1A), beyond approximately 30% than that of previous estimations (86.01% versus 52.2% or 58.6%) [9,10]. This discrepancy is possible because the samples were mainly derived from diseased ducks (Table 1). These ducks frequently show obvious liver pathology. Thus, it is not surprising that we could discover a much higher DHBV prevalence (Fig 1A). Besides, lack of DHBV vaccination may also contribute to a high DHBV infection rate, as there is currently no licenced vaccine for DHBV [20]. Furthermore, insufficient antiviral immune response to DHBV is generally implicated in its chronic infection, which is also linked to its high prevalence [8,21,22]. Regarding the question of which factors dominantly contribute to high DHBV infections, we know little about its infection biology as the virus was largely neglected. In the future, more in-depth investigations will be required. Additionally, our study found that the infection rate of DHAV reached 54.55% (Fig 1A), with the majority being DHAV-3. This result aligns with the current prevalence pattern of DHAV in China [23]. Similarly, studies conducted in South Korea also revealed that DHAV-3 is the most prevalent genotype [24]. This suggests that DHAV-3 widely dominates current duck farms.

Besides the higher prevalence of DHBV (123/143) (Fig 1A), we unexpectedly found a much higher DHBV-related co-infections with all other four types of duck hepatitis viruses (Fig 2A), especially the coinfection between DHBV and DHAV (Fig 2D). In humans, a similar pattern of coinfection between hepatitis B virus (HBV) and hepatitis A virus (HAV) is very rare, with only one case reported so far [25]. However, HBV and hepatitis D virus (HDV) coinfections are common [26,27]. This implies a different hepatitis virus co-infection pattern between ducks and humans. Of note, the discovery of high DHBV and DHAV coinfection in veterinary research is new, which may be attributed to the following reasons. First, DHBV and DHAV can be transmitted vertically [22,28]. Besides, it has been reported that the antiviral interferon response triggered by HBV infection is low or insufficient [28]; simultaneously, the DHAV can dramatically inhibit host innate immunity [29,30]. These factors may collectively make the two hepatitis viruses more prone to coinfecting the ducks, as do higher co-infection events.

In duck farms, it has been reported that DHBV can also co-infect with other non-hepatotropic viruses, such as duck circovirus (DuCV) [31]. Beyond coinfection related to hepatotropic virus, coinfection between goose parvovirus and DuCV was also noticed [32]. Thus, there are many patterns of coinfection occurring in duck populations. In addition to these existing patterns, our data further supplement several hepatitis virus-related coinfection patterns. However, the functional impacts of these coinfections between the co-infected viruses and their combined effect on host pathogenesis remains largely unknown. Among different patterns, we thought it was important to investigate the co-infection of DHBV with other hepatitis viruses by studying how one virus affects the viral replication of another and the resulting disease severity. The DHAV and DHBV coinfection should be investigated first. This recommendation is mainly due to the seriousness of DHAV, the high prevalence of DHBV (Fig 1A), and their high coinfection rates (Fig 2A).

In the present study, we provided an epidemiological update of the five types of duck hepatitis viruses. We discovered an emerging problem of DHBV-related coinfections, especially the DHBV and DHAV coinfections. We anticipate our findings will stimulate further research into the biological impacts of DHBV-related coinfection in ducks and their consequence on liver pathogenesis.

## Supporting information

S1 Table Primers used for qPCR detection of five duck hepatitis viruses, as well as bacterial 16S rRNA PCR. (DOCX)

S2 Table CT values from qPCR detection of five hepatitis viruses in 143 liver samples. (DOCX)

S1 Fig Agarose gel electrophoresis analysis of bacterial 16S rRNA amplified from livers. PCR products were run on 1% agarose gel. Positive samples were marked by a “*”.
(TIF)

S2 Fig Standard curve of DHAV-3. Serial 10-fold dilutions of viral RNA standards (DHAV-3: from 2.65 × 10^8^ to 2.65 × 10^2^ copies/μL) were plotted against the threshold cycle (Ct) values. X-axis: The log values of DHAV-3 RNA copies. Y-axis: The corresponding Ct values. The coefficient of determination (R2) and the equation for the regression curve (y) were calculated as Y = −3.906X + 50.536.
(TIF)

S3 Fig Association analysis of DHDV and DHEV-related coinfections. **(A)** The positive sample numbers of co-infected hepatitis viruses with or without DHDV. The impact of DHDV infection on their coinfection with other hepatitis viruses. The student’s T-test was used for statistical analysis. **(B)** The positive sample numbers of co-infected hepatitis viruses with or without DHEV. The impact of DHEV infection on their coinfection with other hepatitis viruses.
(TIF)

S1 raw images Raw gel image corresponding to S1 Fig in the main text. PCR products of 16S rRNA gene amplification using 27F/1492R primers.
(PDF)
