# Supplementary material for: High prevalence of Duck Hepatitis B virus-associated coinfection in Southwest China
Source: PLoS One. 2025 Jun 16;20(6):e0324682. doi: 10.1371/journal.pone.0324682 (PMC12169529; doi:10.1371/journal.pone.0324682)
Supplement: S1 Table — (DOCX) [file pone.0324682.s001.docx]

| **Table S1 Primers used in this study** | | |
| --- | --- | --- |
| **Viruses** | **Forward Primers** | **Reverse Primers** |
| DHAV-1 | CCTAACTCTGCCATTTACATCAACCAC | GGTATCACCTTGATTGTTAGTTGCCATCT |
| DHAV-3 | GCTTGGGGTTATTCCCAAACCC | GCTTGCAATATTTCAGCACCACCTC |
| DHBV | GGCTAGGAGATTGCTTTGGTGGC | GGACTTTGAACGTCTTCTCCCATAGAC |
| DHCV | CACGAACCAACGCCATCGTTC | AGGGACAGCAAGCACCTTACG |
| DHDV | GGACCGGAGTTCACAGCTTACC | GGCCTGGGCAATCCATAGTAGATC |
| DHEV | GGGTGGTAACCCCCTAYCTTACC | CTGCCACCGCTGCTCATC |
| 16s RNA | AGAGTTTGATCCTGGCTCAG | GGTTACCTTGTTACGACTT |
