# Supplementary material for: High prevalence of Duck Hepatitis B virus-associated coinfection in Southwest China
Source: PLoS One. 2025 Jun 16;20(6):e0324682. doi: 10.1371/journal.pone.0324682 (PMC12169529; doi:10.1371/journal.pone.0324682)
Supplement: S2 Table — (DOCX) [file pone.0324682.s002.docx]

**Table S2 CT values from qPCR detection of five hepatitis viruses in 143 liver samples**

| **Sample ID** | **DHAV-1** | **DHAV-3** | **DHBV** | **DHCV** | **DHDV** | **DHEV** |
| --- | --- | --- | --- | --- | --- | --- |
| 1 | 35.38 | 34.51 | 18.25 | N/A | 42.24 | 44.15 |
| 2 | 38.84 | 30.73 | 30.83 | N/A | 35.19 | 36.51 |
| 3 | 37.10 | 37.08 | 35.11 | 43.51 | 37.15 | 34.21 |
| 4 | 37.66 | 37.42 | 33.30 | 39.24 | 39.62 | 35.27 |
| 5 | N/A | 34.66 | 28.73 | 35.08 | 35.91 | 33.70 |
| 6 | N/A | 35.02 | 27.49 | 39.47 | 32.91 | 33.03 |
| 7 | 40.82 | 35.24 | 32.37 | N/A | 33.48 | 35.26 |
| 8 | N/A | 35.09 | 34.44 | 41.84 | 34.52 | 34.87 |
| 9 | 41.02 | 34.20 | 34.98 | 37.96 | 33.19 | 36.00 |
| 10 | N/A | 35.65 | 28.46 | 40.04 | 33.12 | 36.94 |
| 11 | N/A | 35.04 | 28.20 | 37.44 | 32.93 | 35.41 |
| 12 | N/A | 35.40 | 34.86 | 36.74 | 33.78 | 34.75 |
| 13 | 40.32 | 36.08 | 37.40 | 35.47 | N/A | 33.85 |
| 14 | 40.58 | 31.77 | N/A | 39.43 | N/A | 33.31 |
| 15 | 41.85 | 34.42 | 30.06 | 39.33 | 37.15 | 34.36 |
| 16 | N/A | 35.63 | 33.58 | 39.58 | 36.69 | 34.28 |
| 17 | N/A | 35.10 | 31.29 | 44.63 | 34.52 | 34.89 |
| 18 | 42.55 | 33.12 | 34.12 | N/A | 32.51 | 35.03 |
| 19 | 39.27 | 34.26 | 34.34 | 38.25 | 19.12 | 35.52 |
| 20 | 23.42 | 31.31 | 35.55 | 37.67 | 32.52 | 35.08 |
| 21 | N/A | 34.70 | 31.01 | 35.59 | 42.03 | 33.05 |
| 22 | 37.57 | 35.26 | 29.95 | 41.52 | 32.52 | 35.24 |
| 23 | N/A | 36.28 | 29.45 | 34.58 | N/A | 36.56 |
| 24 | N/A | 33.49 | 4.05 | 38.03 | N/A | 34.50 |
| 25 | 33.72 | 28.49 | 9.68 | 36.70 | 43.71 | 36.66 |
| 26 | 39.41 | 27.79 | 26.17 | 34.55 | 33.70 | 36.27 |
| 27 | 36.67 | 28.87 | 26.58 | 38.84 | 37.56 | 35.60 |
| 28 | 37.01 | 35.31 | 29.45 | 38.47 | 35.35 | 36.00 |
| 29 | 39.04 | 30.81 | 32.40 | 40.16 | 38.14 | 32.16 |
| 30 | 38.31 | 31.22 | 26.60 | 39.67 | 43.03 | 36.11 |
| 31 | 39.01 | 34.29 | 30.18 | 34.78 | 32.45 | 33.08 |
| 32 | 42.61 | 34.98 | 33.83 | 39.63 | 32.54 | 34.12 |
| 33 | 39.49 | 34.71 | 31.82 | 38.81 | 32.98 | 34.08 |
| 34 | 34.13 | 35.42 | 33.18 | 38.04 | 33.28 | 33.82 |
| 35 | 36.28 | 37.04 | 32.37 | 40.08 | 36.61 | 43.42 |
| 36 | N/A | 36.89 | 35.18 | 37.50 | 33.97 | 35.01 |
| 37 | 37.51 | 40.86 | 26.43 | N/A | 40.15 | 40.01 |
| 38 | 39.66 | 34.36 | 17.77 | 35.82 | 32.36 | 33.29 |
| 39 | 35.78 | 30.57 | 16.03 | N/A | 34.50 | 34.72 |
| 40 | N/A | 35.23 | 26.65 | N/A | N/A | 37.22 |
| 41 | 39.05 | 33.87 | 30.45 | 41.08 | 33.60 | 34.86 |
| 42 | N/A | 32.30 | 31.36 | 36.34 | 23.50 | 37.56 |
| 43 | N/A | 34.08 | 35.79 | 38.30 | 31.13 | 34.19 |
| 44 | N/A | 35.11 | 33.16 | 40.16 | 34.14 | 35.48 |
| 45 | 37.25 | 30.64 | 27.49 | 41.00 | 37.47 | 36.09 |
| 46 | 40.20 | 35.49 | 33.62 | 42.57 | 33.92 | 35.30 |
| 47 | 40.48 | 35.58 | 29.83 | N/A | 28.58 | 38.26 |
| 48 | N/A | 35.29 | 34.32 | 36.85 | 32.61 | 34.09 |
| 49 | 40.74 | 34.48 | 34.08 | 33.91 | 33.18 | 35.13 |
| 50 | 39.35 | 35.02 | 32.17 | 39.85 | 33.46 | 34.54 |
| 51 | N/A | 34.86 | 31.09 | 40.31 | 34.78 | 35.35 |
| 52 | 39.55 | 30.80 | 31.94 | 41.33 | 22.22 | 36.61 |
| 53 | N/A | 33.25 | 32.83 | 40.00 | 32.05 | 35.81 |
| 54 | N/A | 33.71 | 33.13 | 37.62 | 36.71 | 31.63 |
| 55 | N/A | 34.02 | 35.03 | 35.24 | 33.62 | 32.60 |
| 56 | N/A | 34.14 | 34.77 | 39.07 | 38.78 | 34.77 |
| 57 | 38.99 | 35.49 | 29.41 | 41.18 | 35.48 | N/A |
| 58 | N/A | 35.34 | 33.18 | 39.16 | 36.15 | 32.41 |
| 59 | N/A | 40.17 | 31.79 | N/A | N/A | 41.11 |
| 60 | 38.31 | 36.81 | 26.60 | 42.61 | 29.19 | 35.80 |
| 61 | 38.49 | 33.02 | 28.42 | N/A | 40.42 | 37.36 |
| 62 | 37.85 | 24.89 | 19.61 | 33.23 | 33.21 | 31.45 |
| 63 | 38.41 | 29.90 | 18.75 | 35.54 | 24.91 | 35.25 |
| 64 | 41.77 | 36.53 | 32.93 | 41.11 | 38.80 | 37.91 |
| 65 | 42.72 | 35.24 | 33.03 | 38.77 | 35.88 | 33.01 |
| 66 | 41.63 | 33.06 | 32.40 | 45.58 | 28.97 | 36.03 |
| 67 | 39.45 | 33.00 | 34.59 | 40.72 | 28.76 | 33.27 |
| 68 | 39.36 | 35.85 | 36.65 | 33.47 | 34.03 | 35.11 |
| 69 | N/A | 34.97 | 37.57 | 37.45 | 32.14 | N/A |
| 70 | 38.52 | 36.87 | 36.86 | 42.39 | 35.06 | 37.52 |
| 71 | N/A | 34.00 | 41.31 | 41.31 | 32.39 | 32.92 |
| 72 | N/A | 38.63 | 37.18 | 37.02 | N/A | 34.70 |
| 73 | N/A | 32.68 | 40.17 | 41.53 | 36.02 | 36.24 |
| 74 | 43.84 | 33.90 | 39.16 | 33.47 | 30.02 | 32.25 |
| 75 | 38.16 | 39.15 | 34.53 | N/A | N/A | 41.32 |
| 76 | 41.48 | 33.67 | 34.56 | 40.72 | 32.15 | 32.68 |
| 77 | 41.02 | 33.86 | 33.36 | 35.54 | 42.79 | 38.85 |
| 78 | 44.53 | 33.03 | 30.03 | 42.56 | 25.40 | 34.62 |
| 79 | N/A | 30.63 | 23.66 | 41.03 | 27.40 | 37.94 |
| 80 | 41.18 | 27.56 | 28.48 | 36.08 | 25.04 | 36.97 |
| 81 | N/A | 33.01 | 24.38 | 35.05 | 34.27 | 34.24 |
| 82 | 39.32 | 28.17 | 21.90 | 33.30 | 26.89 | 33.04 |
| 83 | 39.29 | 29.03 | 22.92 | 42.06 | 31.01 | 36.72 |
| 84 | 37.27 | 36.16 | 30.16 | 41.62 | 39.82 | 38.06 |
| 85 | N/A | 36.09 | 16.33 | 36.85 | 35.75 | 34.66 |
| 86 | 38.29 | 34.96 | 32.06 | 35.08 | 36.45 | 37.26 |
| 87 | 45.63 | 33.85 | 38.51 | 37.42 | 31.81 | 34.30 |
| 88 | 41.30 | 33.21 | 34.18 | 37.22 | 32.37 | 36.94 |
| 89 | N/A | 34.03 | 45.35 | 41.33 | N/A | 35.15 |
| 90 | 38.52 | 37.01 | 33.21 | 39.41 | 34.65 | 35.45 |
| 91 | 3.82 | 33.43 | 33.86 | 36.88 | 38.60 | 25.57 |
| 92 | 42.44 | 32.82 | 39.81 | 35.27 | 36.01 | 37.60 |
| 93 | 42.33 | 33.69 | 31.47 | 44.90 | 21.91 | 36.27 |
| 94 | N/A | 32.85 | 37.12 | 39.30 | 23.75 | N/A |
| 95 | N/A | 33.69 | 32.15 | 41.44 | 35.64 | 37.68 |
| 96 | 40.45 | 34.55 | 8.31 | N/A | 38.05 | 36.12 |
| 97 | 38.31 | 34.20 | 7.71 | N/A | 38.84 | 37.00 |
| 98 | 39.68 | 37.83 | 6.07 | 42.05 | 36.62 | N/A |
| 99 | 39.01 | 34.55 | 18.09 | N/A | 35.16 | 38.12 |
| 100 | 41.96 | 36.05 | 7.69 | 44.18 | 36.51 | 36.70 |
| 101 | 40.03 | 38.45 | 17.10 | 45.03 | 36.80 | 37.26 |
| 102 | N/A | 36.12 | 7.83 | 35.19 | 31.17 | 35.32 |
| 103 | 40.38 | 35.25 | 20.83 | 41.10 | 38.48 | 36.06 |
| 104 | 41.70 | 31.06 | 29.50 | 36.62 | N/A | 35.26 |
| 105 | 43.39 | 34.00 | 27.31 | 45.49 | N/A | 33.68 |
| 106 | 41.14 | 36.42 | 33.43 | 33.78 | 39.23 | 35.91 |
| 107 | 43.02 | 35.32 | 31.85 | 45.53 | 39.67 | 34.81 |
| 108 | 43.52 | 34.59 | 32.81 | 37.25 | 33.44 | 34.61 |
| 109 | 43.62 | 33.25 | 43.60 | 38.25 | N/A | 34.83 |
| 110 | 43.55 | 36.57 | 27.64 | N/A | 30.83 | 34.28 |
| 111 | 40.65 | 33.11 | 28.16 | 38.00 | 35.06 | 34.38 |
| 112 | 42.64 | 31.79 | 21.98 | 37.86 | 39.77 | 35.26 |
| 113 | 39.76 | 34.43 | 32.32 | 39.61 | 36.53 | 33.14 |
| 114 | 38.58 | 36.25 | 31.67 | N/A | 37.54 | 34.80 |
| 115 | 39.26 | 39.58 | 22.48 | N/A | N/A | N/A |
| 116 | 28.76 | 6.03 | 4.22 | 17.37 | 6.48 | 23.45 |
| 117 | N/A | 35.23 | 30.60 | 30.85 | 26.89 | 28.04 |
| 118 | 35.01 | 43.55 | 15.49 | 44.09 | N/A | N/A |
| 119 | 38.00 | 34.22 | 17.06 | 45.38 | 35.87 | 33.59 |
| 120 | 37.05 | 32.95 | 25.75 | 45.84 | 37.70 | 35.24 |
| 121 | 39.95 | 32.72 | 19.77 | 43.26 | 37.92 | 35.44 |
| 122 | 38.93 | 44.20 | 29.29 | N/A | N/A | N/A |
| 123 | 41.30 | 31.51 | 19.43 | 37.08 | 37.01 | 35.61 |
| 124 | 37.06 | 40.45 | 25.82 | 36.35 | N/A | N/A |
| 125 | 39.01 | 35.06 | 30.49 | N/A | 43.04 | 35.79 |
| 126 | 40.44 | 37.15 | 5.51 | 29.11 | N/A | N/A |
| 127 | 39.10 | 37.25 | 4.07 | 38.36 | 39.53 | 35.53 |
| 128 | 38.83 | 36.99 | 27.59 | N/A | N/A | N/A |
| 129 | 38.67 | 37.99 | 25.46 | 44.08 | N/A | N/A |
| 130 | 38.35 | 37.83 | 29.41 | 35.86 | N/A | N/A |
| 131 | 38.11 | 37.21 | 29.06 | 29.09 | N/A | 36.88 |
| 132 | 40.49 | 37.08 | 27.30 | 34.05 | N/A | 39.25 |
| 133 | 37.31 | 37.72 | 24.81 | N/A | N/A | 38.33 |
| 134 | 40.36 | 31.99 | 30.13 | 44.16 | N/A | N/A |
| 135 | 39.75 | 36.23 | 26.87 | N/A | N/A | N/A |
| 136 | 40.43 | 36.16 | 26.23 | 40.15 | 36.58 | 36.22 |
| 137 | 38.72 | 36.05 | 27.90 | N/A | 39.03 | 38.22 |
| 138 | 37.86 | 36.65 | N/A | N/A | 44.12 | 39.55 |
| 139 | 40.66 | 35.20 | 23.36 | 43.81 | 35.36 | 34.64 |
| 140 | 38.90 | 39.68 | 27.30 | N/A | 41.15 | 33.93 |
| 141 | 37.16 | 36.91 | 23.57 | N/A | 42.43 | N/A |
| 142 | 36.40 | 20.47 | 29.11 | N/A | 44.37 | 35.08 |
| 143 | N/A | 36.79 | 25.33 | N/A | 40.36 | 39.70 |
| SUM^a^ | 11 | 75 | 123 | 14 | 59 | 53 |

^a^SUM refers to the total number of five duck hepatitis virus positive samples. For the duck hepatitis virus samples, a CT value greater than 35 is considered negative, while for DHAV-3, a CT value greater than 37 is considered negative.
