# Supplementary figures and images for: High prevalence of Duck Hepatitis B virus-associated coinfection in Southwest China
Source: PLoS One. 2025 Jun 16;20(6):e0324682. doi: 10.1371/journal.pone.0324682 (PMC12169529; doi:10.1371/journal.pone.0324682)

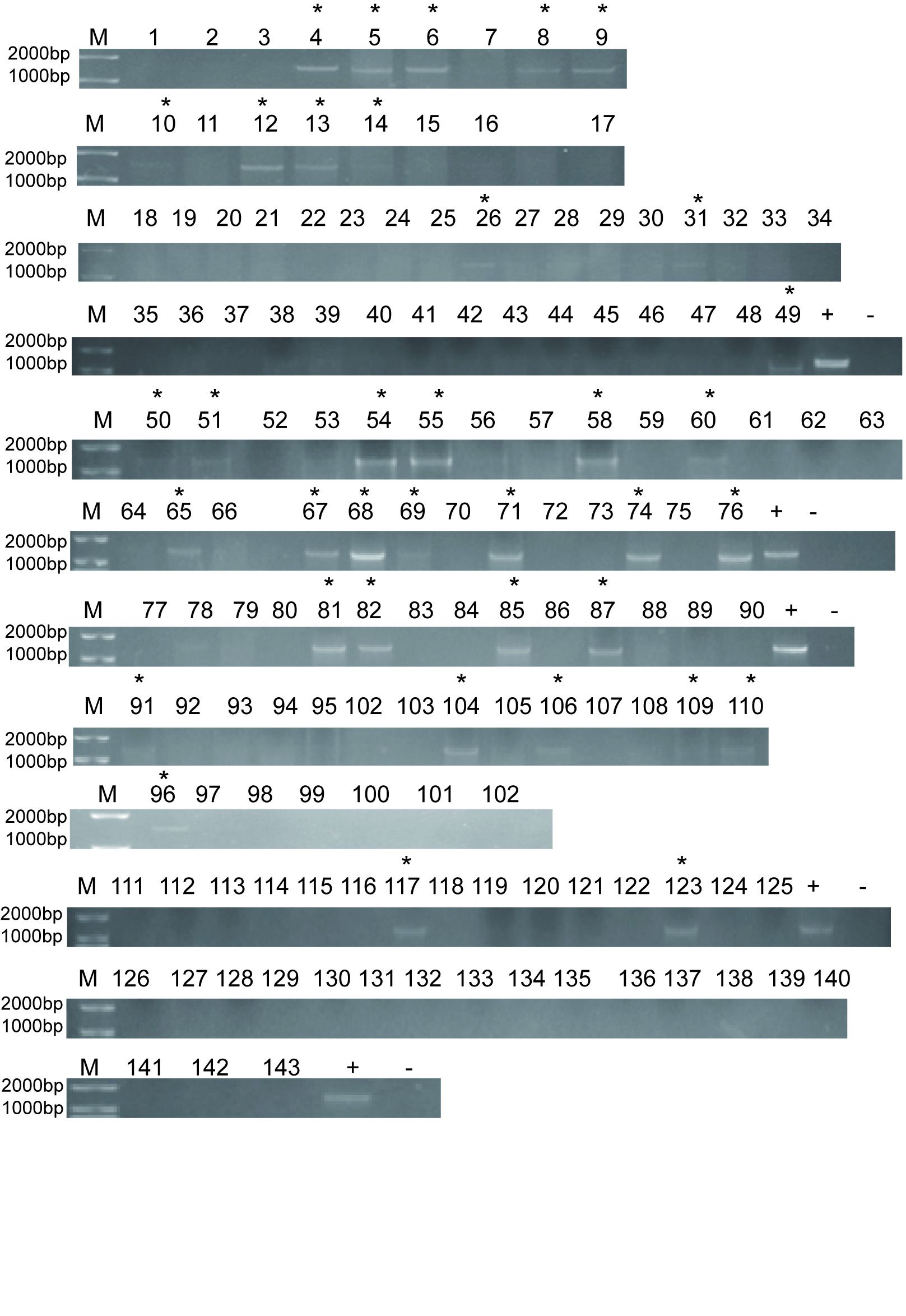

Supplement: S1 Fig — PCR products were run on 1% agarose gel. Positive samples were marked by a “*”. (TIF) [file pone.0324682.s003.tif]

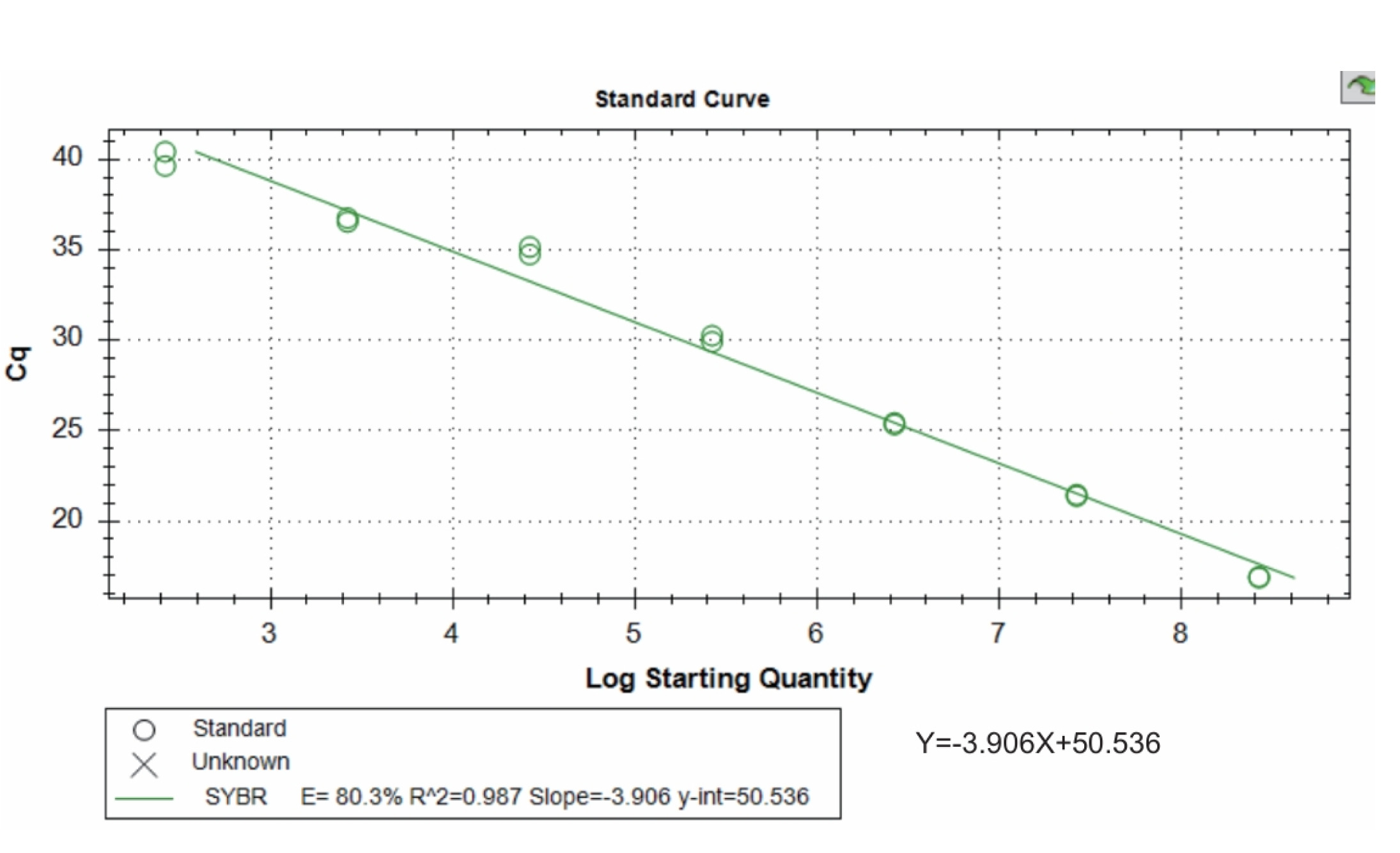

Supplement: S2 Fig — Serial 10-fold dilutions of viral RNA standards (DHAV-3: from 2.65 × 10^8^ to 2.65 × 10^2^ copies/μL) were plotted against the threshold cycle (Ct) values. X-axis: The log values of DHAV-3 RNA copies. Y-axis: The corresponding Ct values. The coefficient of determination (R2) and the equation for the regression curve (y) were calculated as Y = −3.906X + 50.536. (TIF) [file pone.0324682.s004.tif]

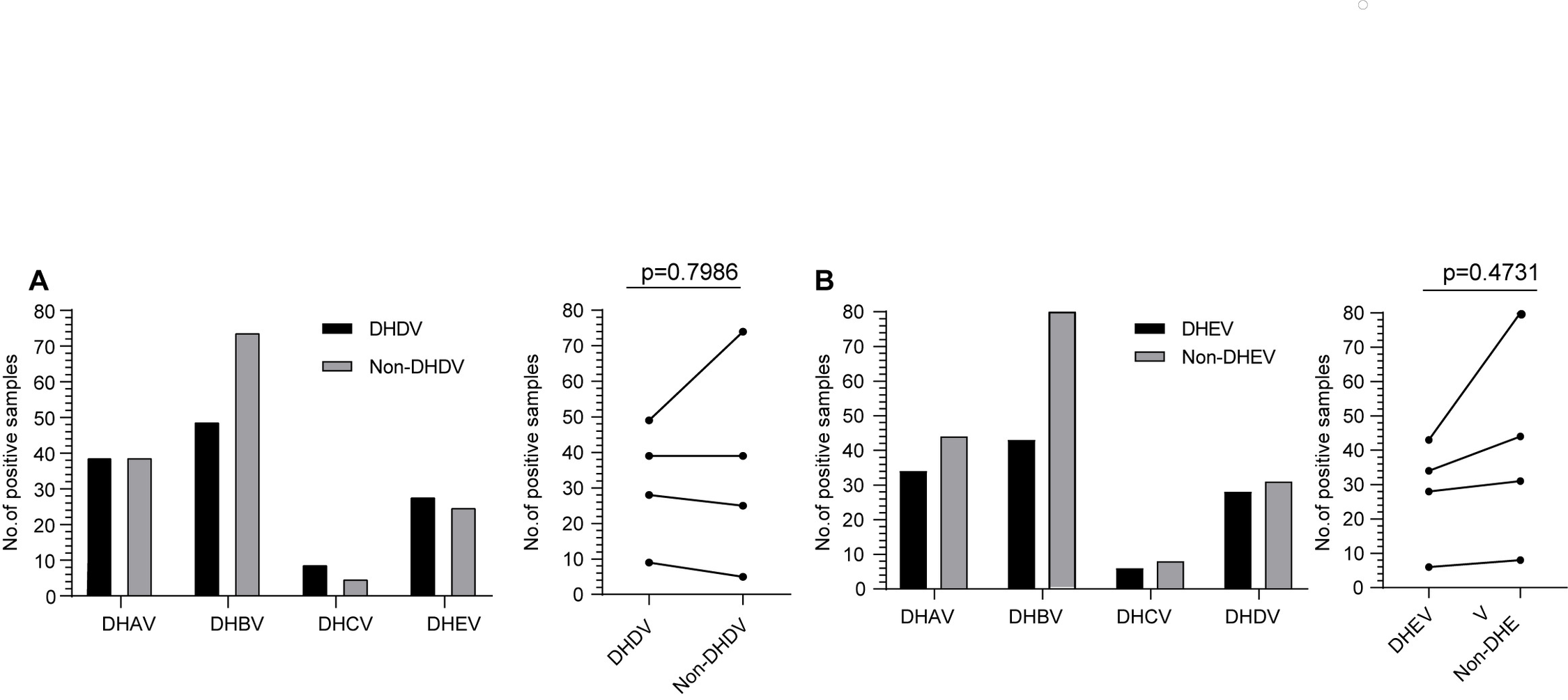

Supplement: S3 Fig — (A) The positive sample numbers of co-infected hepatitis viruses with or without DHDV. The impact of DHDV infection on their coinfection with other hepatitis viruses. The student’s T-test was used for statistical analysis. (B) The positive sample numbers of co-infected hepatitis viruses with or without DHEV. The impact of DHEV infection on their coinfection with other hepatitis viruses. (TIF) [file pone.0324682.s005.tif]
