# Supplementary material for: High prevalence of Duck Hepatitis B virus-associated coinfection in Southwest China
Source: PLoS One. 2025 Jun 16;20(6):e0324682. doi: 10.1371/journal.pone.0324682 (PMC12169529; doi:10.1371/journal.pone.0324682)
Supplement: S1 raw images — PCR products of 16S rRNA gene amplification using 27F/1492R primers. (PDF) [file pone.0324682.s006.pdf]

M 1 2 3 4 5 6 7 8 9
M 10 11 12 13 14 15 16 X 17

2000bp
1000bp
750bp
500bp
250bp
100bp

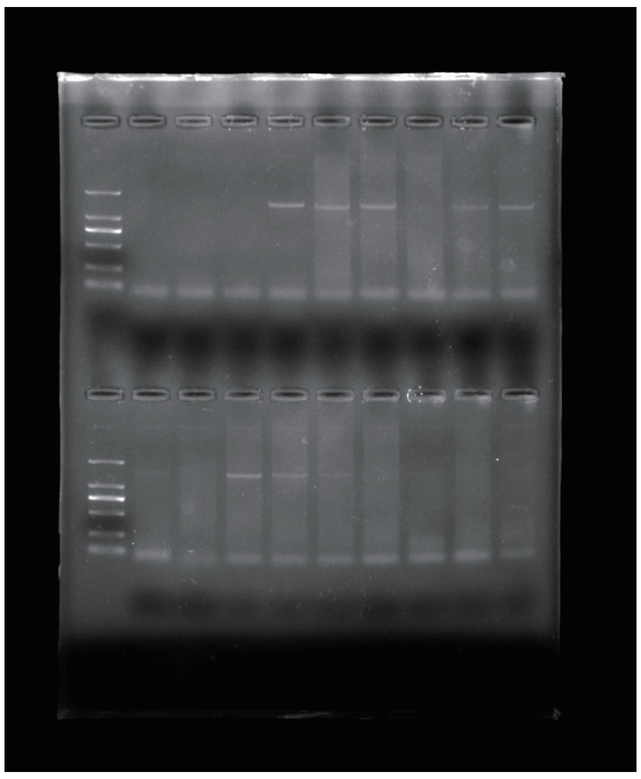

M 18 19 20 21 22 23 24 25 26 27 28 29 30 31 32 33 34

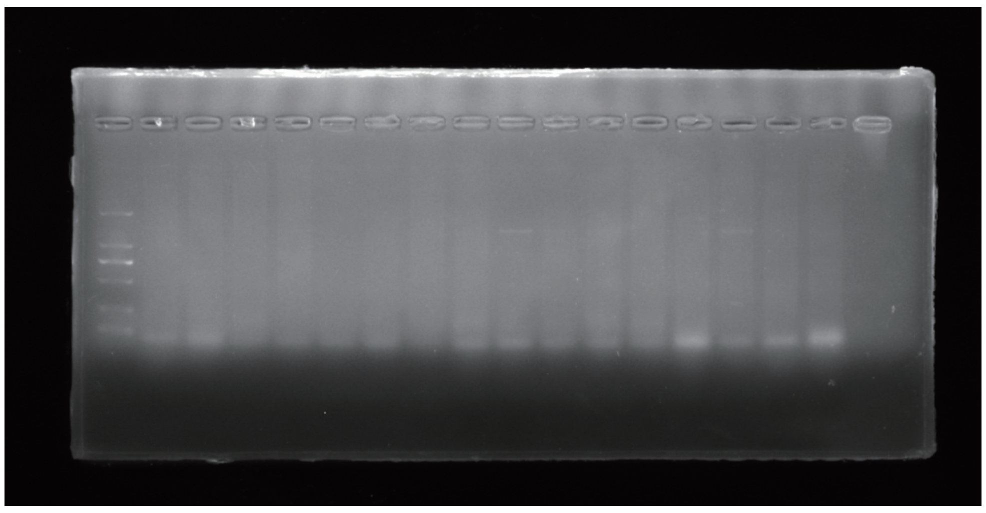

M 35 36 37 38 39 40 41 42 43 44 45 46 47 48 49 + -
M 50 51 52 53 54 55 56 57 58 59 60 61 62 63 + -
M X X X X X X X X X X X X X X X X
M X X X X X X X X X X X X X X X X X

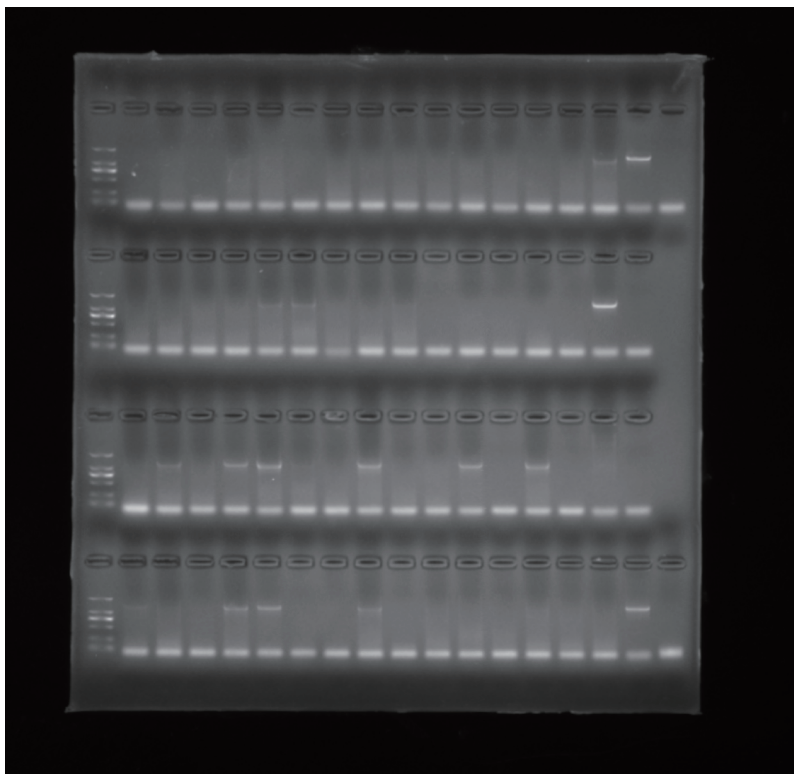

M 64 65 66 X 67 68 69 70 71 72 73 74 75 76 + -
M 77 78 79 80 81 82 83 84 85 86 87 88 89 90 + -
M 91 92 93 94 95 102103104105106107108109110 X X

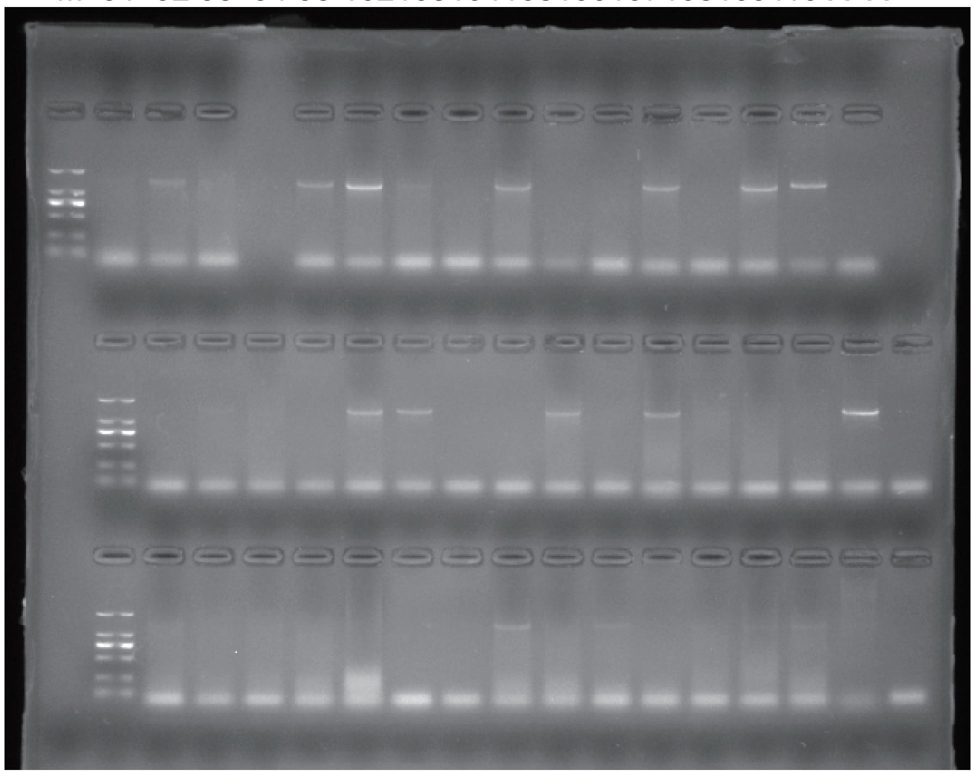

M 96 97 98 99 100 101 102 X

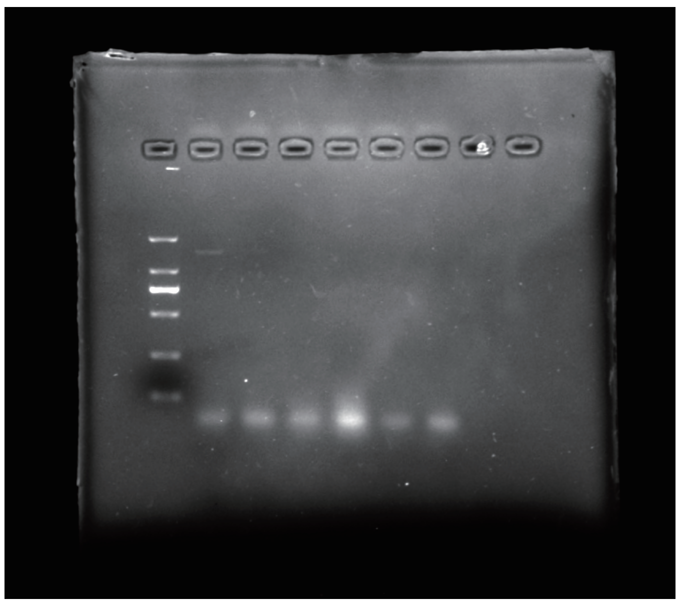

M 111 112 113 114 115 116 117 118 119 120 121 122 123 124 125 + -

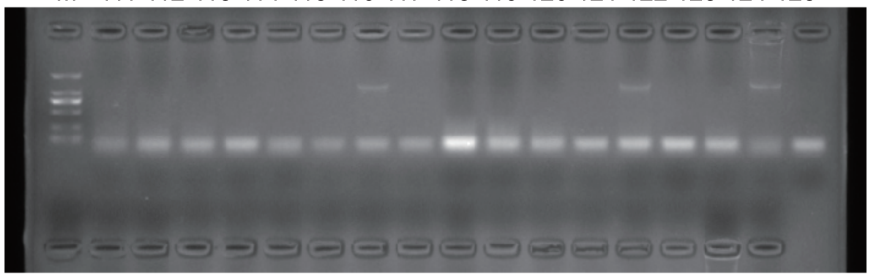

M X X X X X X X X X X X X X X X X X X X X X M X X X
M X X X X X X X X X X X X X X X X X
M 126 127 128 129 130 131 132 133 134 135 136 137 138 139 140 X X
M 141 142 143 + - X X X X X X X X X X X

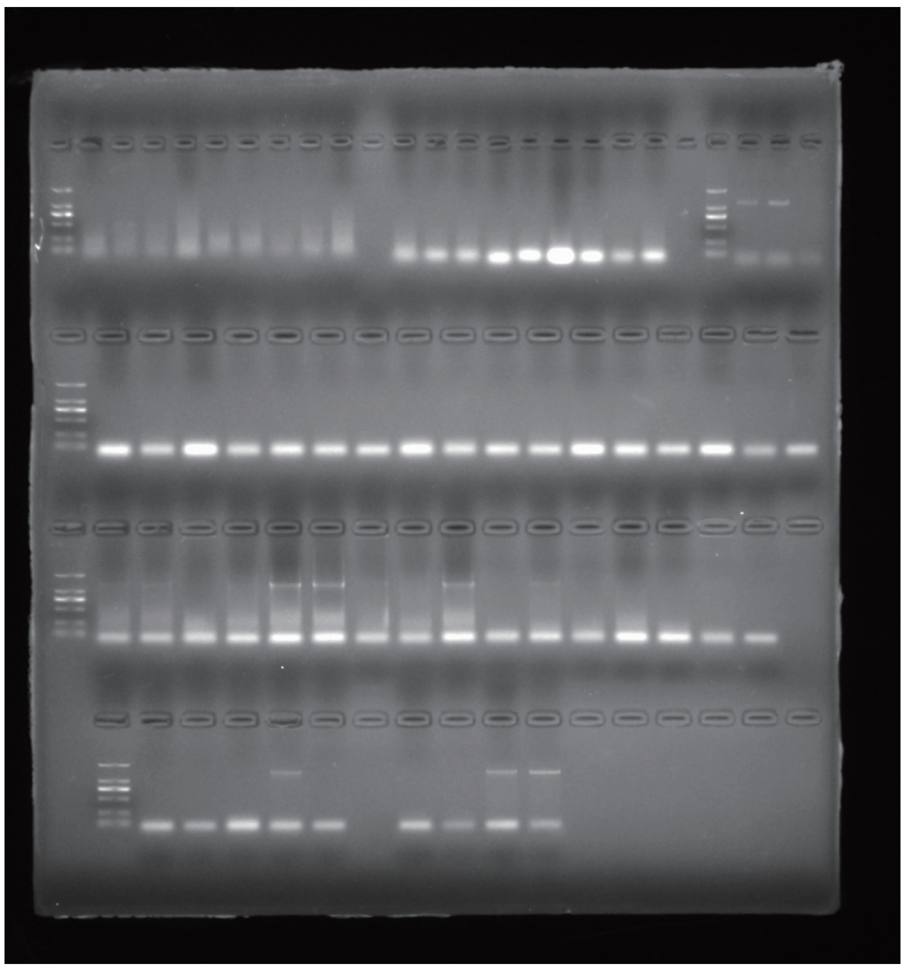

All the samples in this document are PCR products analyzed by gel electrophoresis. Genomic DNA was extracted from duck liver tissue, and specific primers targeting the 16S rRNA gene were used for PCR amplification. The PCR products were then analyzed by gel electrophoresis using 1.2% agarose gel. The results were analyzed with Image Lab software, and images were captured using the same software. The lane numbers correspond to the sample identifiers, ranging from 1 to 144.

The electrophoresis images shown in the DPF are derived from the original gel images in Supplementary Material S1. The sample numbers displayed in the DPF directly correspond to those labeled in the images of Supplementary Material S1.

The following are unedited, raw gel electrophoresis images supporting the detection of bacterial infections in samples using 16S rRNA primers:

- **Marker (M)**: The molecular weight marker used in this study is 2000 bp, with bands corresponding to 2000 bp, 1000 bp, 750 bp, 500 bp, 250 bp, and 100 bp from top to bottom.
- **Expected Size**: The expected size of the 16S rRNA amplicon is approximately 1542 bp, and the observed bands align with this expected size.
- **Sample Labels**: Each gel image is labeled at the top with the sample sequence.
- **Controls**:

o The symbol "+" indicates the positive control, where *Escherichia coli* culture was used.

o The symbol "-" indicates the negative control, where ddH_2O was added during PCR.

- **Unrelated Lanes**: The symbol "X" denotes lanes unrelated to this experiment, and their results are not included in the main text.

Additional Notes:

☒ **Samples 111-125**: The gel images for these samples may appear cropped, but this is because the entire gel was not captured during imaging. Only the portion containing the samples was collected.
